# Genomic Insight into Three Marine *Micromonospora* sp. Strains from the Gulf of California

**DOI:** 10.1128/MRA.01673-18

**Published:** 2019-07-11

**Authors:** Luis Contreras-Castro, Luis A. Maldonado, Erika T. Quintana, Lorena Carro, Hans-Peter Klenk

**Affiliations:** a Laboratorio de Bioprospección en Actinobacterias, Escuela Nacional de Ciencias Biológicas (ENCB), Instituto Politécnico Nacional (IPN), Mexico City, Mexico; b Facultad de Química, Universidad Nacional Autónoma de México (UNAM), Mexico City, Mexico; c School of Natural and Environmental Science, Newcastle University, Newcastle upon Tyne, United Kingdom; d Departamento de Microbiología y Genética, Universidad de Salamanca, Salamanca, Spain; University of Arizona

## Abstract

Three actinomycete strains, designated BL1, BL4, and CV4, were isolated from sediment samples from the Gulf of California in 2009 together with nearly 300 other actinobacteria. Genome mining and analysis of their ∼6.4-Mb sequences confirmed the bioprospecting potential of these three bacteria belonging to the genus *Micromonospora*.

## ANNOUNCEMENT

The genus *Micromonospora*, class *Actinobacteria*, contained over 80 validly described species (1) at the time of writing. Although several species have terrestrial origin, they have also been associated with marine environments (2), sea sands (3), near-shore sediments (4), deep-sea sediments (5, 6), and marine sponges (7). *Micromonospora* spp. show well-developed, branched, substrate mycelium, with nonmotile spores and usually absent aerial mycelium, although there are recent reports opposing this view (8).

Micromonosporae show 16S rRNA similarity values within the range of 96.7% to 99% (2), making the description of putative novel species difficult without full-genome sequences. Micromonosporae have also been found to be a promising source of biosynthetic metabolites (9).

Strains BL1, BL4, and CV4 were part of a previous report on the isolation of almost 300 actinobacteria (10) from the Gulf of California and Gulf of Mexico. Single colonies were selected for DNA extraction from isolates cultured on ISP2 medium (11). Genomes were sequenced by MicrobesNG using the Illumina MiSeq sequencing platform. Genomic DNA libraries were prepared using a Nextera XT library prep kit (Illumina, San Diego, CA, USA). DNA quantification and library preparation were carried out on a Hamilton Microlab STAR automated liquid-handling system. Pooled libraries were quantified using the Kapa Biosystems library quantification kit for Illumina on a Roche LightCycler 96 quantitative PCR (qPCR) machine. Libraries were sequenced using a 250-bp paired-end protocol. The reads were trimmed using Trimmomatic v.0.38, and the quality was assessed using in-house scripts combined with the following software: SAMtools, BedTools, and BWA-MEM (12–15). *De novo* assembly was performed using SPAdes 3.1.1 using default parameters (16). Contigs were annotated with the NCBI Prokaryotic Genome Annotation Pipeline (17).

Genome characteristics are summed up in Table 1. EzTaxon analysis of the 16S rRNA (18) identified that the three species belonged to the genus *Micromonospora*; BL1 is related to Micromonospora tulbaghiae with 100% 16S rRNA gene similarity, while BL4 and CV4 are related to Micromonospora coriariae, both with 99.72% 16S rRNA gene similarity. It is worth noting that recent reviews of actinobacterial phylogeny (19) have proven that 16S rRNA phylogeny is not enough for a correct identification even of the genus in these closely related genera.

**TABLE 1:** Characteristics of the *Micromonospora* sp. isolate genomes

Isolate	Median insert size (bp)	Mean coverage (×)	Mean coverage excluding 0s (×)	No. of reads	No. of reads with insert size >300 bp	No. of contigs	*N*_50_ value	Genome size (Mb)	G+C content (%)	No. of open reading frames	No. of tRNAs/rRNAs
BL1	496	31.9427	31.9761	592,434	416,541	422	33,132	6.46	72.9	6,153	52/6
BL4	487	41.0063	41.0232	732,201	512,449	283	53,042	6.41	72.2	6,036	49/6
CV4	532	36.5438	36.5923	631,879	502,335	454	34,445	6.37	72.2	6,122	49/7

An average nucleotide identity (ANI) matrix was generated using different *Micromonospora* species (20). The ANI value between BL4 and CV4 was 98.9%, suggesting that both isolates belong to the same species and its most related species is *M. coriariae* DSM 44875^T^, a bacterium isolated from the plant Coriaria myrtifolia (21), with an ANI of 93.2%, which is below the species delimitation percentage of 95%. This suggests that BL4 and CV4 may constitute a novel species (22). As for BL1, the ANI value with *M. tulbaghiae* DSM 45142^T^ was 96.2%. This result, along with the EZTaxon identification, shows that BL1 should be identified as *M. tulbaghiae*, a species first isolated from leaves of the South African plant Tulbaghia violacea (23). The fact that these micromonosporae were recovered from marine sediments and that BL1 has been reported to produce aerial hyphae, an unusual feature on micromonosporae (8), could imply different ecological niches for *Micromonospora* spp. and roles in aquatic environments.

Genome mining using antiSMASH 3.0 (24) and the Web tool NaPDoS (25) predicted the production of bleomycin, lymphostin, phosphonoglycan, actinomycin, alnumycin, epothilone, spinosad, syringomycin, and sioxanthin biosynthetic clusters among the three genomes. The xantholipin cluster was found only in BL1 and BL4, alkyl-*O*-dihydrogeranyl-methoxyhydroquinone on BL1 and CV4, and desferrioxamine B on both BL4 and CV4. Exclusive clusters were azicemicin, leinamycin, lobosamide, nocathiacin, pentalenolactone, terramycin, curacin, tyrocidin, and oxazolomycin for BL1, ochronotic pigment for BL4, and pradimicin for CV4.

### Data availability.

This whole-genome shotgun project has been deposited in GenBank under the accession no. RCVK00000000, RCVJ00000000, and RCVI00000000 and in the Sequence Read Archive under the accession no. SRR8727741, SRR8727780 and SRR8727783. The versions described in this paper are the first versions, RCVK01000000, RCVJ01000000, and RCVI01000000.
